# The Prion Basis of Progressive Neurodegenerative Disorders

**DOI:** 10.1155/2023/6687264

**Published:** 2023-02-14

**Authors:** Tvisha Joshi, Nidhi Ahuja

**Affiliations:** ^1^Dublin High School, 8151 Village Pkwy, Dublin, California 94568, USA; ^2^University of California, Berkeley Extension 1995 University Ave, Berkeley, California 94704, USA

## Abstract

The discovery of proteinaceous infectious agents by Prusiner in 1982 was sensational. All previously known pathogens contained nucleic acids, the code of life, that enabled them to reproduce. In contrast, the proteinaceous agents of disease, called prion proteins (PrP), lacked nucleic acids and propagated by binding to the functional, endogenous form of cellular prion protein (referred to as PrP^C^) and altering its conformation to produce the infectious disease-causing misfolded protein (referred to as PrP^Sc^). The accumulation and aggregation of these infectious prion proteins within the brain cause destruction of neural tissue and lead to fatal spongiform encephalopathies. In this review, we present the molecular pathology of prion-based diseases. These insights are of particular importance since the principles of prion pathogenesis apply to other neurodegenerative diseases such as Alzheimer's disease, Huntington's disease, Parkinson's disease, and amyotrophic lateral sclerosis. Collectively, the global prevalence of these diseases is rapidly increasing while effective therapies against them are still lacking. Thus, the need to understand their etiology and pathogenesis is urgent, and it holds profound implications for societal health.

## 1. Historical Background

For nearly half a century, baffled physicians had watched their patients suffer from a rapidly progressive deterioration of brain functions [1, 2]. This neurodegenerative disorder that ultimately killed the patients was named as Creutzfeldt-Jakob disease (CJD). It was observed that while CJD destroyed the brain of patients and dementia took over, the immune system was slow to respond. In 1959, Klatzo et al. recognized the similarities between the pathologies of CJD and another neurodegenerative disease called kuru that was prevalent in the natives of New Guinea [3]. In that same year, Hadlow noticed how signs of kuru were similar to those of scrapie [4]. It was believed at the time that scrapie was caused by a “slow virus,” as proposed by Sigurdsson in 1954 while he was conducting studies on scrapie sheep in Iceland [5]. The transmissibility of kuru and CJD was established by Gajdusek et al. and their colleagues, who had passaged these diseases to chimpanzees [6]. Gordon had been previously shown to be transmissible in sheep [7]. For many years, it was incorrectly assumed that these three neurodegenerative diseases, namely, CJD, kuru, and scrapie, resulted from infections of slow or latent viruses but we now know that all of these diseases are caused by prions.

## 2. Prion Diseases in Humans

### 2.1. Creutzfeldt-Jakob Disease

In humans, CJD has been the most studied prion-based disease. It is a rapidly progressing neurodegenerative disorder that affects approximately one person in a population of a million every year. In the United States of America, this statistic equates to roughly 330 new cases in a year, and among these, about 85% cases are of sporadic CJD that occur without a discernible environmental source of infection. Sporadic CJD causes progressive neurologic dysfunction in multiple areas of the brain that may be associated with alteration of personality or behavior, insomnia, anorexia, depression, involuntary movements, severe cognitive impairment, and death within a few months [8]. In about 5% of patients with sporadic CJD, the onset of neurologic dysfunction is so abrupt that it may be confused with a stroke. Familial CJD, unlike sporadic CJD, results from mutations of the gene for PrP, and accounts for up to 15% cases. The features of familial CJD vary according to the underlying mutation, but in general, there is an earlier age of onset in familial cases along with a more prolonged duration of illness. Contaminated medical instruments or biological material such as corneal grafts, human dura mater grafts, and human pituitary-derived hormones have been documented to result in a few cases of acquired CJD known as iatrogenic CJD. Signs and symptoms of iatrogenic CJD depend on the route of inoculation. When the route of inoculation is through the central nervous system, then the clinical presentation of the disease is like that of sporadic CJD, whereas with a peripheral route of inoculation an ataxic syndrome results. In 1996, a variant form of acquired CJD (vCJD) was identified in people who had eaten contaminated beef that was derived from cows suffering from bovine spongiform encephalopathy. Besides ingestion of contaminated beef, vCJD can also be spread through contaminated surgical instruments, transfusion of tainted blood, transplants, or exposure to infected tissues and their derivatives. In vCJD, the cases initially present psychiatric symptoms such as depression and withdrawal for months prior to the development of neurologic signs [9]. The terminal stages of vCJD appear similar to those of sporadic CJD.

### 2.2. Kuru

The word Kuru in Fore means “to shake,” and it is used to describe a slow progressing fatal infection of the brain. It is a transmissible spongiform encephalopathy that is associated with deposits of kuru amyloid plaques in the cerebellum, thalamus, and cerebral cortex that are followed by severe gliosis of astrocytes, neuronal lesions, and spongiform changes within the brain. Kuru became an epidemic in New Guinea between 1950 and 1970 because of the endocannibalistic funeral rituals practiced by the native Fore population. The investigations of the Fore people by anthropologists suggested that kuru had begun in the region between 1900 and 1920 [10]. While the incubation period for kuru can be several decades, death usually occurs within months of the onset of symptoms. Initially, Kuru presents with progressive trembling, an unsteady gait, and dysarthria. With time, muscle tremors become more severe, ataxia worsens, and the patient loses voluntary muscle control. Unlike other prion diseases, severe dementia does not occur in kuru [11]. Currently, this disease is considered eradicated due to the elimination of tribal cannibalism.

### 2.3. Gerstmann–Sträussler–-Scheinker (GSS) Disease

Alterations in the PrP gene sequence can result in heritable prion-based diseases. All cases of GSS result from such inherited mutations of the PrP gene that are passed on to the next generation in an autosomal dominant manner. Those who possess a disease-causing mutation in PrP gene can eventually develop signs and symptoms because of neurodegeneration; however, the progression and the overall severity of their disorder can vary depending upon on the underlying mutation. A common symptom that appears early is a progressive loss of coordination that may be seen as unsteady gait, difficulty in walking, and inept movement of limbs. As the disease progresses, other symptoms start appearing such as problems associated with cognition, language, memory, and behavior. In GSS, PrP^Sc^ can be found in many parts of the brain, including the cerebellum and thalamus, and this isoform can transmit the disease to experimental animals [12].

### 2.4. Fatal Familial Insomnia (FFI)

The D178N mutation of PrP gene is associated with the development of a progressive sleep disorder called FFI. In patients who have the PrP D178N mutation along with a methionine at the 129^th^ position of PrP, a fatal sleep disorder presents itself with an average onset around the age of 50 and death occurring within a year of onset of symptoms [13]. In the brains of these patients, deposits of PrP^Sc^ plaques are confined mainly to the thalamus. In contrast, when the PrP D178N mutation is combined with a valine at the 129^th^ position of PrP, it results in the development of familial CJD. These patients experience dementia, and deposits of PrP^Sc^ plaques can be seen throughout the brain upon postmortem [14]. Clinical signs of FFI include altered sleep–wake cycles with decreased vigilance, dysfunction of the autonomic system that presents as systemic hypertension and irregular breathing, and motor manifestations such as diplopia, dysphagia, dysarthria, ataxia, and abasia. Sleep–wake and vigilance abnormalities are characterized by insomnia, hallucinations, and confusion. The clinical signs can vary according to the mutation at codon 129.

## 3. PrP Biology

The gene encoding for PrP is present on the human chromosome #20. After its transcription, the translation of the entire reading frame occurs from a single RNA exon. This removes the possibility that PrP^Sc^ plaque production or development of prion diseases results from alternative splicing of its RNA [15]. During development, the PrP mRNA expression is stringently regulated, but in adults, this mRNA is constitutively expressed with its highest levels being produced in the neurons [16]. Upon translation, the mRNA produces a polypeptide that is 253 amino acids long and has several key features including an amino-terminal signal peptide, a conserved hydrophobic region in the center followed by another hydrophobic region at the carboxy-terminus that serves as an attachment site for a glycosylphosphatidylinositol (GPI) anchor. Another interesting feature of this polypeptide is its repeat sequences that are comprised of one copy of the nonapeptide PQGGGGWGQ and four copies of the octapeptide PHGGGWGQ. Majority of PrP^C^ are found attached to the outer face of the cell membrane by their C-terminal GPI anchors. The pathway followed by PrP^C^ for this targeting is same as that of other membrane-bound proteins which involves synthesis on the rough ER and sorting through Golgi before reaching the cell surface. Along this path, PrP^C^ is post-translationally processed to allow for the removal of a signal peptide from the amino terminus and of a stretch of 23 amino acids from the carboxy-terminal, a disulfide bridge formation, the addition of oligosaccharides at the amino terminus, and of a GPI-anchor at the carboxy-terminus [17]. The processed protein is a ∼208 amino acid long chain that folds into two domains of roughly equal size. Studies have demonstrated that the attachment of a GPI anchor along with the number of oligosaccharides attached to PrP can vary [18]. Many different cell types, including neurons, secrete PrP^C^ without its anchor to such an extent that it can accumulate to 10% of total PrP^C^ in brain extracts. The absence of GPI anchor does not automatically cause PrP misfolding, but it has been observed that PrP anchored on the cell surface is important in controlling neuronal toxicity [19]. Anchored PrP^C^ does not stay on the cell surface long after its delivery there, but rather it recycles between the cell membrane and an endocytic vesicle [20].

Prion protein is highly conserved among mammals, so it is surprising that animals lacking PrP^C^ develop and behave normally [21]. Interestingly, animals that do not express prion proteins do not succumb to prion disease. Several cellular functions have been proposed for PrP^C^. These include regulation of myelination, copper metabolism, cell communication, cell migration, cell differentiation, cell survival, circadian rhythm, and inhibition of Bax-mediated apoptosis [22–27].

## 4. Prion Propagation

A small number of PrP^Sc^ presented to the host cell can corrupt endogenous PrP^C^ and convert them all to PrP^Sc^. During this transition, the shift in the secondary structure of the protein becomes obvious: *α*-helices predominate the secondary structure of PrP^C^, whereas *β*-sheets predominate in PrP^Sc^ [28]. The spontaneous *α*-to-*β* transition in the structure of prion protein is rare and the most fundamental event that defines the pathogenesis of prion diseases. This structural change is associated not only with loss of function but also with aggregation and pathological amyloid assembly via templating mechanisms. The initial trigger for amyloid assembly may not always be exogenous PrP^sc^. Mutations that destabilize the structure of endogenous prion protein can promote amyloid formation and lead to prion diseases with sporadic or genetic origin.

The molecular mechanism that converts PrP^C^ to PrP^Sc^ is not conclusively known. However, several models have been proposed, including a direct conversion mechanism, whereby each protein in the PrP^Sc^ state can bind to PrP^C^ to form a heterodimer and then through this interaction convert PrP^C^ to PrP^Sc^. Another model suggests that a cofactor may unfold PrP^C^ to a partially unfolded PrP^*∗*^ state that may facilitate formation of the heterodimer with PrP^Sc^ [29]. Either way, subsequent addition of PrP^C^ to the heterodimer results in fibril formation, its elongation that ultimately leads to amyloid assembly. Depending on its conformational stability, the growing fibril may break and release new templates that spread and continue to support the chain reaction of prion protein misfolding and aggregation in different parts of the brain.

Although the precise intracellular location of PrP^Sc^ formation is not yet known but the conversion of the PrP^C^ to PrP^Sc^ is postulated to begin on the cell surface and continue within caveolae-like domains along the endocytic trafficking pathway [30]. After this conversion, PrP^Sc^ starts accumulating in the lysosomes and in the late endocytic vesicles of the affected cells [31]. This intracellular accumulation of PrP^Sc^ induces vacuolation of neurons and hypertrophy of reactive astrocytes that eventually kills the neurons [32]. At this point, PrP^Sc^ begins to accumulate extracellularly. PrP^Sc^ polymerizes to form amyloid plaques that leads to more death of neurons, progressive destabilization of neuronal networks, and spongiform degeneration of the brain that is a hallmark of prion diseases.

## 5. Prion-Like Neurodegenerative Diseases

The clinical presentation of human prion diseases bears striking resemblance to the pathological hallmarks of many other commonly known progressive neurodegenerative disorders [33]. The common signs include memory loss, dementia, difficulties with movement, loss of brain function, and changes in behavior and personality. At a molecular level, these neurodegenerative diseases are characterized by accumulation of misfolded protein deposits that correlate with progressive loss of neurons and accumulation of amyloid lesions in the brain. The multimeric amyloid assemblies of misfolded proteins implicated in these diseases include amyloid-*β* plaques in Alzheimer's disease, *α*-synuclein Lewy bodies in Parkinson's disease, huntingtin protein aggregates in Hungtington's disease, and multiple protein aggregates in amyotrophic lateral sclerosis [34–37]. The endogenous proteins implicated in each of these diseases use similar mechanisms for misfolding, polymerization, and propagation. A common theme seen in these proteins is that they all misfold to conformations that are rich in *β*-sheets [38]. In all these cases, the misfolded proteins lead to chain reactions whereby they act as templates to corrupt the conformation of normal proteins [39]. These misfolded proteins aggregate to form amyloids that the disrupt function either by directly harming nearby cells, and/or by sequestering essential proteins that are unable to execute their role within the body. The ability of these proteins to propagate by the process of conversion followed by aggregation, allows the disease to be initiated by a small number of misfolded proteins that set-in motion a rapidly progressing disorder. Since the normal and misfolded isoforms of the proteins have the same sequence, and since the host is already tolerant to the normal isoform, these diseases do not elicit a severe immune response.

## 6. Conclusion

It has been 40 years since proteinaceous infectious particles were discovered. They have been studied a lot since then but the mechanisms by which they cause neurodegeneration have not been fully uncovered. Pathology of other neurodegenerative diseases where misfolded aggregated proteins are implicated is also poorly understood. Understanding how PrP^C^ misfolds into PrP^Sc^ and how these corrupted proteins form toxic aggregates may open new avenues to develop effective therapies not just for prion diseases but also for other common neurodegenerative disorders too.

## References

[1] Creutzfeldt H. G. (1920). Über eine eigenartige herdförmige erkrankung des zentralnervensystems (vorläufige mitteilung). *Zeitschrift für die gesamte Neurologie und Psychiatrie*.

[2] Jakob A. (1921). Über eigenartige erkrankungen des zentralnervensystems mit bemerkenswertem anatomischen befunde. *Zeitschrift für die gesamte Neurologie und Psychiatrie*.

[3] Klatzo I., Gajdusek D. C., Zigas V. (1959). Pathology of kuru. *Laboratory investigation; a journal of technical methods and pathology*.

[4] Hadlow W. J. (1959). scrapie and kuru. *The Lancet*.

[5] Sigurdsson B. (1954). RIDA, A chronic encephalitis of sheep: with general remarks on infections which develop slowly and some of their special characteristics. *British Veterinary Journal*.

[6] Gajdusek D. C., Gibbs C. J., Alpers M. (1966). Experimental transmission of a kuru-like syndrome to chimpanzees. *Nature (London)*.

[7] Gordon W. S., Gordon W. S. (1946). “Advances in veterinary research. *Advances in Veterinary Research*.

[8] Roods R., Gajdusek D. C., Gibbs C. J. (1973). The clinical characteristics of transmissible creutzfeldt-jakob disease. *Brain*.

[9] Will R. G., Ironside J. W., Zeidler M. (1996). A new variant of creutzfeldt-jakob disease in the UK. *The Lancet (North American Edition)*.

[10] Glasse R. M., lindenbaum S., prusiner S. B., collinge J., powell J., anderton B. (1992). Fieldwork in the south fore: the process of ethnographic enquiry. *Prion Diseases of Humans and Animals*.

[11] Liberski P. P., Gajos A., Sikorska B., Lindenbaum S. (2019). Kuru, the first human prion disease. *Viruses*.

[12] Nonno R., Angelo Di Bari M., Agrimi U., Pirisinu L. (2016). Transmissibility of gerstmann-sträussler-scheinker syndrome in rodent models: new insights into the molecular underpinnings of prion infectivity. *Prion*.

[13] Goldfarb L. G., Petersen R. B., Tabaton M. (1992). Fatal familial insomnia and familial creutzfeldt-jakob disease: disease phenotype determined by a DNA polymorphism. *Science*.

[14] Goldfarb L., Haltia M., Brown P. (1991). New mutation in scrapie amyloid precursor gene (at codon 178) in Finnish creutzfeldt-jakob kindred. *The Lancet*.

[15] Basler K., Oesch B., Scott M. (1986). Scrapie and cellular PrP isoforms are encoded by the same chromosomal gene. *Cell*.

[16] Chesebro B., Race R., Wehrly K. (1985). Identification of scrapie prion protein-specific mRNA in scrapie-infected and uninfected brain. *Nature (London)*.

[17] Stahl N., Baldwin M. A., Burlingame A. L., Prusiner S. B. (1990). Identification of glycoinositol phospholipid-linked and truncated forms of the scrapie prion protein. *Biochemistry*.

[18] Borchelt D. R., Rogers M., Stahl N., Telling G., Prusiner S. B. (1993). Release of the cellular prion protein from cultured cells after loss of its glycoinositol phospholipid anchor. *Glycobiology*.

[19] Caughey B., Baron G. S., Chesebro B., Jeffrey M. (2009). Getting a grip on prions: oligomers, amyloids, and pathological membrane interactions. *Annual Review of Biochemistry*.

[20] Shyng S.-L., Huber M. T., Harris D. A. (1993). A prion protein cycles between the cell surface and an endocytic compartment in cultured neuroblastoma cells. *Journal of Biological Chemistry*.

[21] Büeler H., Fischer M., Lang Y. (1992). Normal development and behaviour of mice lacking the neuronal cell-surface PrP protein. *Nature (London)*.

[22] Westergard L., Christensen H. M., Harris D. A. (2007). The cellular prion protein (PrP C): its physiological function and role in disease. *Biochimica et Biophysica Acta, Molecular Basis of Disease*.

[23] Pinheiro L. P., Linden R., Mariante R. M. (2015). Activation and function of murine primary microglia in the absence of the prion protein. *Journal of Neuroimmunology*.

[24] Bounhar Y., Zhang Y., Goodyer C. G., LeBlanc A. (2001). Prion protein protects human neurons against bax-mediated apoptosis. *Journal of Biological Chemistry*.

[25] Hornshaw M. P., Mcdermott J. R., Candy J. M., Lakey J. H. (1995). Copper binding to the N-terminal tandem repeat region of mammalian and avian prion protein: structural studies using synthetic peptides. *Biochemical and Biophysical Research Communications*.

[26] Collinge J., Whittington M. A., Sidle K. C. L. (1994). Prion protein is necessary for normal synaptic function. *Nature (London)*.

[27] Besnier L. S., Cardot P., Da Rocha B. (2015). The cellular prion protein PrPc is a partner of the wnt pathway in intestinal epithelial cells. *Molecular Biology of the Cell*.

[28] Pan K. M., Baldwin M., Nguyen J. (1993). Conversion of alpha-helices into beta-sheets features in the formation of the scrapie prion proteins. *Proceedings of the National Academy of Sciences*.

[29] Cohen F. E., Pan K. M., Huang Z., Baldwin M., Fletterick R. J., Prusiner S. B. (1994). Structural clues to prion replication. *Science*.

[30] Taraboulos A., Scott M., Semenov A., Avrahami D., Laszlo L., Prusiner S. B. (1995). Cholesterol Depletion and Modification of COOH-Terminal Targeting Sequence of the Prion Protein Inhibit Formation of the Scrapie Isoform. *Journal of Cell Biology*.

[31] Taraboulos A., Serban D., Prusiner S. B. (1990). Scrapie prion proteins accumulate in the cytoplasm of persistently infected cultured cells. *Journal of Cell Biology*.

[32] DeArmond S. J., Mobley W. C., DeMott D. L., Barry R. A., Beckstead J. H., Prusiner S. B. (1987). Changes in the localization of brain prion proteins during scrapie infection. *Neurology*.

[33] Gomez-Gutierrez R., Morales R. (2020). The prion-like phenomenon in alzheimer’s disease: evidence of pathology transmission in humans. *PLoS Pathogens*.

[34] Trojanowski J. Q., Goedert M., Iwatsubo T., Lee V. M. Y. (1998). Fatal attractions: abnormal protein aggregation and neuron death in Parkinson’s disease and lewy body dementia. *Cell Death and Differentiation*.

[35] Beyreuther K., Masters C. L. (1991). Amyloid precursor protein (APP) and ΒZA4 amyloid in the etiology of alzheimer’s disease: precursor-product relationships in the derangement of neuronal function. *Brain Pathology*.

[36] Arrasate M., Finkbeiner S. (2012). Protein aggregates in huntington’s disease. *Experimental Neurology*.

[37] Blokhuis A. M., Groen E. J. N., Koppers M., van den Berg L. H., Pasterkamp R. J. (2013). Protein aggregation in amyotrophic lateral sclerosis. *Acta Neuropathologica*.

[38] Glenner G. G., Wong C. W. (1984). Alzheimer’s disease: initial report of the purification and characterization of a novel cerebrovascular amyloid protein. *Biochemical and Biophysical Research Communications*.

[39] Jaunmuktane Z., Brandner S. (2020). Invited review: the role of prion‐like mechanisms in neurodegenerative diseases. *Neuropathology and Applied Neurobiology*.

